# The Asthma COPD Overlap Syndrome: ACOS. Epidemiology and Historical Perspective

**Published:** 2017

**Authors:** Maarten van den Berge

**Affiliations:** Department of Pulmonary diseases, University Medical Center Groningen, University of Groningen, Groningen, the Netherland.

Asthma and COPD are both highly prevalent chronic lung diseases with a high personal and economic impact. Asthma usually starts at young agewith variable symptoms of wheezing, cough, dyspneu, and bronchial hyperresponsiveness. The airflow obstruction in asthma is often fully reversible after treatment with a bronchodilator. In contrast, COPD usually starts after the age of 40 years in smokers and ex-smokers who develop chronic symptoms of dyspnea, cough and sputum production and display chronic airflow obstruction that is not fully reversible after bronchodilator treatment. In their pure forms, it is easy to distinguish between asthma and COPD. However, it is well recognized in clinical practice that many patients have features compatible with both diseases. To describe this, international asthma and COPD guidelines have recently introduced the term ACOS (Asthma COPD overlap syndrome). Thus far, the underlying mechanisms of ACOS and its appropriately treatment remain largely unclear, because these patients have been systematically excluded from clinical studies.

On the long-term, a subset of up to 20% of asthma patients develops a fixed airflow obstruction (1,2). Interestingly, Fabbri et al demonstrated that the type of inflammation in asthma patients with fixed airway obstruction differs from that seen in COPD (3). They showed that asthma patients irreversible airflow obstruction had significantly more eosinophilic inflammation measured in blood, sputum and bronchoalveolar lavage fluidcompared to patients with COPD with a similar degree of airflow obstruction (3). Interestingly, patients with asthma and irreversible airflow obstruction had a greater rate of lung function decline compared to an asthmatic control group with fully reversible airflow obstruction during a follow-up period of 5 years (4). Their rate of decline was similar to that observed in COPD (4). Importantly, higher sputum eosinophil counts predicted lung function decline in patients with asthma and irreversible airflow obstruction, whereas increasednumbers of sputum neutrophils were associated with lung function decline in COPD. Currently, these adult asthmatics with irreversible airflow obstruction are often labelled as COPD and unjustly denied treatment with ICS (5). The introduction of ACOS will lead to a better recognition of these patients so that this is now prevented. In addition, a better phenotyping in COPD may help to identify those COPD patients who benefit from ICS treatment, for example those with bronchodilator reversibility, bronchial hyperresponsiveness or eosinophilic airway inflammation.

Although bronchodilator reversibility and bronchial hyperresponsiveness are frequently considered to be hallmarks of asthma, they can occur in up to 50% of patients with COPD as well (6). Bleecker et al showed that the improvement in post-bronchodilator FEV_1_ after 8 weeks’ treatment with fluticasone/salmeterol 250/50 μg b.i.d. was significantly greater in COPD patients with (n=161) versus without (n=197) bronchodilator reversibility (7). This is in agreement with the findings of Kitaguchi et al who found a significantly larger improvement in FEV_1_ after 2–3 months of ICS treatment in COPD patients with versus without bronchodilator reversibility, their mean improvements in FEV_1_ being 359 ml and 168 ml respectively (8).

Two further studies did not demonstrate a difference in ICS responsiveness between COPD patients with and without bronchodilator reversibility, but these studies were small and hampered by a lack of power (9,10).

It has been argued that BHR is not of pathophysiological importance in COPD as it would merely reflect a lower pre-challenge FEV_1_ (11). However, this does not appear to be the case, since it was shown in a multivariate regression analysis that a more severe BHR in COPD is independently associated with airway inflammation as reflected by the number of neutrophils, lymphocytes and macrophages in induced sputum and bronchial biopsies (12). One small study showed that COPD patients who exhibited BHR to the indirect stimulus mannitol (n=7) had a significantly greater improvement in FEV_1_ after 3 months’ treatment with ICS compared to COPD patients without BHR to mannitol (n=30). However, this contrasts with the findings of Rutgers *et al* who did not find any improvement in FEV_1_ after 6 weeks’ treatment with budesonide 1600 μg daily in COPD patients with BHR to both methacholine and another indirect stimulus, adenosine 5’-monophosphate (13).

Finally, there is an increasing amount of evidence that the presence of eosinophilic inflammation in sputum and blood predicts a favorable response to ICS treatment in COPD with fewer exacerbations and improved in FEV_1_, at least over a period of up to 12 months (14,15). In this context, our recent findings are also of interest (16). We evaluated genes, previously reported to be associated with Th2-high asthma in two independent cohorts of patients with COPD (16). The 100 genes most up-regulated in the airway epithelium in Th2 high asthma as compared to Th2 low asthma/healthy controls were summarized into a single Th2 composite score using a principal component analysis (PCA) projection algorithm (16). COPD patients with a higher Th2 composite score had a more severe airflow obstruction and displayed asthmatic features, i.e. increased eosinophilic inflammation in their blood and bronchial biopsies, and bronchodilator reversibility. Moreover, they had a favorable treatment response: after both short-(6-month) and long-term (30-month) treatment with inhaled fluticasone with or without added salmeterol: they experienced more improvement in hyperinflation, measured with body plethysmography. These findings are promising as they show that the presence and extent of ‘Th2-driven eosinophilic inflammation’ is a useful biomarker to guide the diagnosis of asthma, COPD, or ACOS. Future longitudinal studies are now needed to better define the clinical implications of ACOS with respect to the long-term outcome and treatment of ACOS and its sub-phenotypes compared to only asthma or COPD.
